# A Quality Initiative for Reducing Postoperative Hypothermia for Neonatal Intensive Care Unit Surgical Patients

**DOI:** 10.1097/pq9.0000000000000318

**Published:** 2020-07-07

**Authors:** Jessica A. Cronin, Lamia Soghier, Kara Ryan, Christine Shen, Sopnil Bhattarai, Sohel Rana, Rahul Shah, Eugenie Heitmiller

**Affiliations:** From the * Division of Anesthesiology, Pain and Perioperative Medicine, Children’s National Hospital, George Washington University, Washington, D.C.; † Division of Neonatology, Children’s National Hospital, George Washington University, Washington, D.C.; ‡ Division of Quality and Safety, Children’s National Hospital, George Washington University, Washington, D.C.; § Center for Surgical Care, Children’s National Hospital, George Washington University, Washington, D.C.

## Abstract

**Introduction:**

The association between hypothermia in the neonatal intensive care unit (NICU) patients and morbidity and mortality is well described. Neonates are at higher risk of perioperative hypothermia when compared to older children. Previous studies showed that quality improvement tools reduced postoperative hypothermia in NICU patients, but none showed sustained improvement at incidence rates of <10%. As a single institution, we aimed to reduce the percentage of postoperative temperatures < 36°C in NICU patients from 10% to 6% over 6 months and sustain for 6 months.

**Methods:**

An interdisciplinary team created a key driver diagram and implemented interventions, including monthly reporting of postoperative hypothermia incidence to the anesthesiologists, individual feedback sessions with the anesthesiologists, use of a perioperative checklist, and continuous axillary temperature monitoring of the infant throughout the perioperative period. Data were collected retrospectively using a chart review of electronic medical records. The primary outcome was the percentage of hypothermic patients (T < 36°C) based on the first postoperative temperature taken in the NICU. We tracked this measure using a statistical control chart and evaluated it using Plan-Do-Study-Act cycles.

**Results:**

From February 1, 2016 to May 30, 2018, data were collected for 554 patients (pre-intervention: 242 and post-intervention: 312). The percentage of surgical patients who returned to the NICU hypothermic decreased from 9.7% to 2.5% (*P* < 0.002)—a change sustained for greater than 12 months.

**Conclusions:**

Quality improvement tools are useful in reducing postoperative hypothermia in NICU surgical patients and in maintaining these results.

## INTRODUCTION

The association between hypothermia in the neonatal intensive care unit (NICU) patients and morbidity and mortality is well described. Hypothermia (body temperature <36°C) increases the risk of metabolic acidosis, increases pulmonary and systemic vascular resistance, reduces cardiac output, and increases the risk of hypoventilation.^1,2^ Previous research showed that infants with hypothermic temperatures upon admission to the NICU had an increased rate of mortality, significant brain injury, severe retinopathy of prematurity, bronchopulmonary dysplasia, necrotizing enterocolitis, sepsis, and prolonged ventilation.^3,4^ Large adult studies suggest that hypothermic surgical patients have increased incidences of wound infection, myocardial infarction, need for transfusion, and greater need for assisted ventilation than normothermic patients.^5–8^ While large studies have not described this issue in the pediatric surgical population, it is reasonable to believe adverse perioperative events may also occur in hypothermic infants.

Neonates are at greater risk of hypothermia compared to older children and adults, as they have a higher ratio of surface area to body weight, reduced body fat, and an underdeveloped ability to shiver.^2^ This risk increases further when NICU patients require a procedure in the operating room (OR).^1^ General anesthesia causes hypothermia through a reduction in metabolic heat generation, redistribution of body heat due to vasodilation, and anesthetic-induced central inhibition of thermoregulation.^1^ The OR setting contributes to hypothermia because of the cool ambient temperature and the patient’s increased exposure to the environment.^9^ Morehouse et al found that NICU surgical patients who underwent procedures in the OR were more likely to be hypothermic than those who underwent procedures in the NICU. They also found that hypothermic patients required more cardiac and respiratory interventions than normothermic counterparts.^10^

Given the high risk of hypothermia in surgical patients and its associated sequelae, the Centers for Medicaid and Medicare Services tracks postoperative hypothermia and incentivizes a reduction in its incidence. U.S. News and World Report uses this metric for their hospital rankings.^11,12^ Previous research indicates improved clinical outcomes with better temperature management in adults.^13^

Checklists have been used successfully in the delivery room to decrease hypothermic temperatures for premature infants being admitted to the NICU.^14^ Engorn et al^15^ showed that the use of a checklist helped reduce postoperative hypothermia in NICU patients. Furthermore, the Children’s Hospitals Neonatal Consortium STEPP-IN initiative published results showing that a multi-institutional quality improvement project reduced postoperative hypothermia from 20.3% to 10.5%.^16^ However, no studies have demonstrated sustained improvement below 10%. We aimed to reduce the percentage of postoperative temperatures <36°C in NICU patients from 10% to 6%, an approximately 40% reduction, over 6 months from May 1 to November 30, 2017, and sustain for 6 months, until May 1, 2018. This project aims to decrease postoperative hypothermia in all NICU patients without restriction based on age, weight, or surgery timing.

## METHODS

**Fig. 1.**
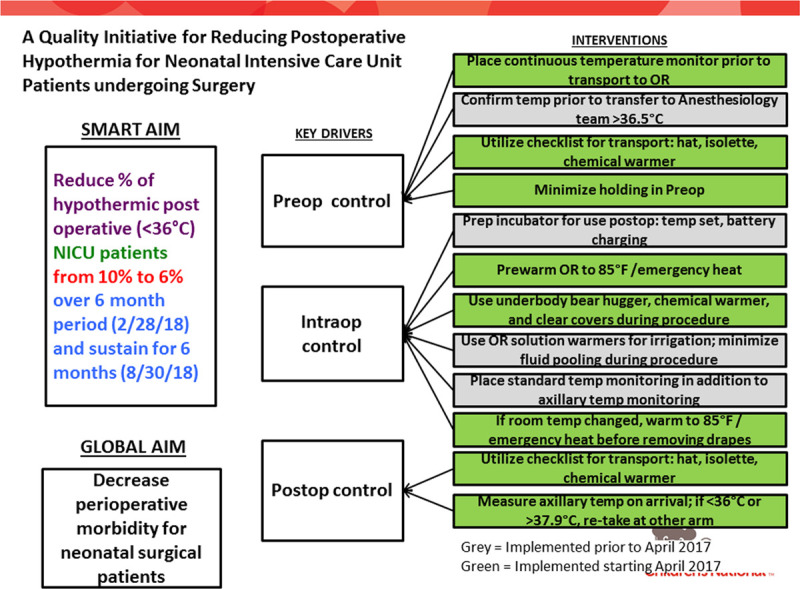
Key driver diagram. Frontline Clinical teams were engaged at each step. Specifically, NICU nurses and anesthesiology teams were engaged and performed the interventions that involved preoperative temperature control. Anesthesiology team, perioperative nurses, and surgical techs were engaged to implement intraoperative temperature control interventions. Finally, NICU nurses and anesthesiology teams were actively engaged to improve postoperative temperature control.

**Fig. 2.**
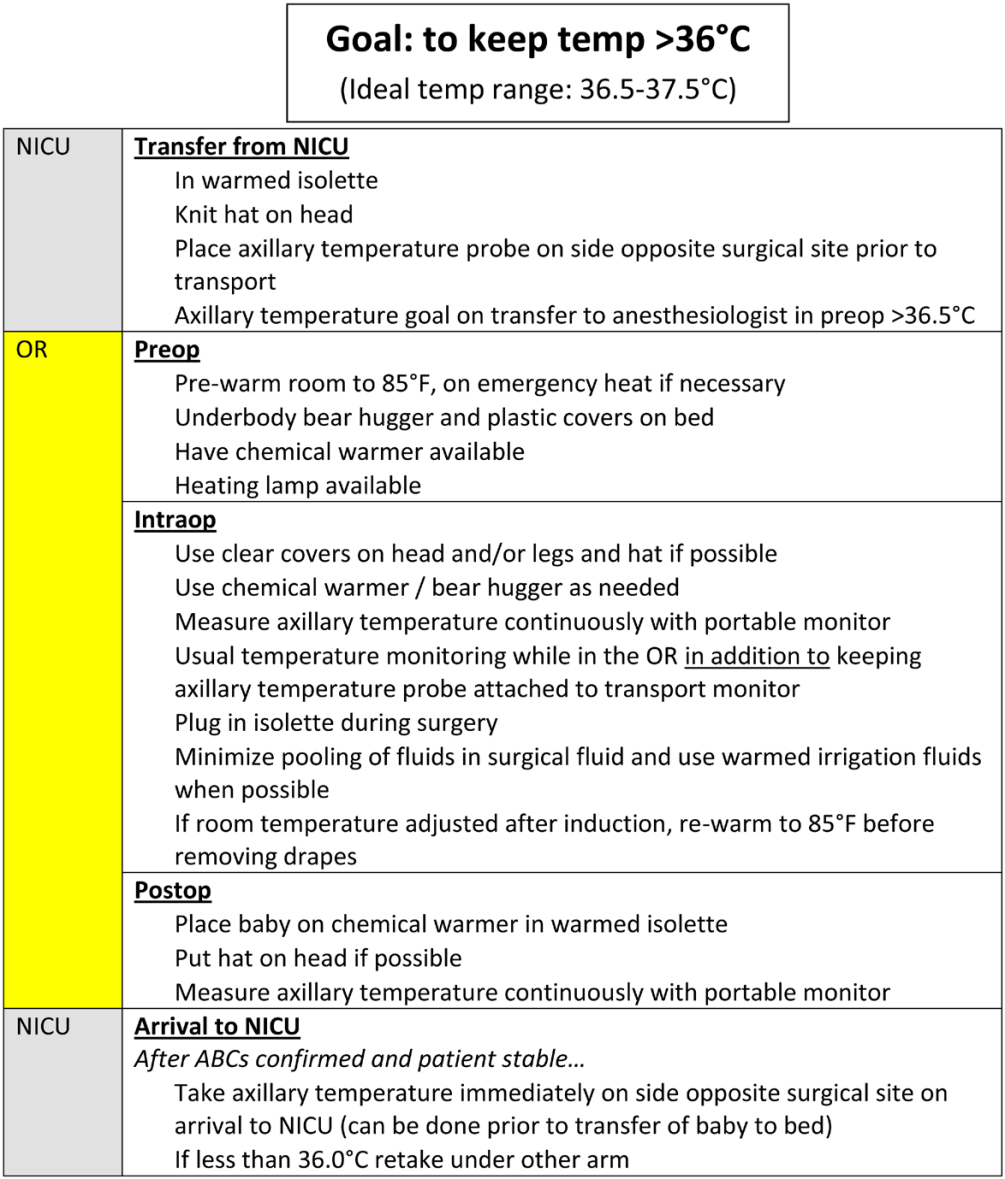
OR-NICU temperature checklist.

Children’s National Hospital is an urban, tertiary-care, free-standing, academic children’s hospital. It has a level 4 NICU with more than 800 admissions per year. Within a quality improvement program at this institution, an interdisciplinary team that included clinical staff from neonatology, anesthesiology, and biomedical engineering created a key driver diagram (Fig. 1). The team implemented this initiative on April 1, 2017, focusing on multiple interventions, including:

(1) Monthly reporting of incidence of postoperative hypothermia to all members of the anesthesiology department starting April 2017.
(2) Individual feedback session with the anesthesiologist whenever postoperative hypothermia occurred starting May 2017. Before interventions, the cases of postoperative hypothermia (n = 23) were distributed across 15 anesthesiologists, so anesthesiologists did not cluster cases. Nonetheless, these discussions addressed obstacles to maintaining normothermia in the specific patient and interventions that may be helpful in the future. Further, this intervention was intended to increase engagement among anesthesiologists.
(3) Use of a perioperative checklist to remind clinicians about the use of chemical warmers, hats, warming of the OR, and other useful tools to reduce hypothermia (Fig. 2) starting August 2017. This checklist is readily available online, where other perioperative algorithms, guidelines, and pathways are also stored for our institution and are frequently accessed.
(4) Continuous axillary temperature monitoring of the infant throughout the perioperative period from departure from NICU, throughout the entire period in the OR, up until return to NICU starting August 2017. When this project began, NICU nurses transported the patient to the preoperative area before surgery, while the anesthesiology team transported the patient back to the NICU postoperatively. Multiple interventions to improve compliance with this intervention included providing a temperature probe to the specific NICU nurse taking care of the surgical patient on the day of surgery and weekly meetings with NICU nurse educators to discuss compliance. Just-in-time training, in the form of reminder calls to the individual NICU nurse about using temperature probes, occurred before transport to the preoperative area. In October 2017, the anesthesiology team started to transport the patient from the NICU to the OR; however, the NICU nurse was still responsible for placing the temperature probe on the infant before transport.

**Fig. 3.**
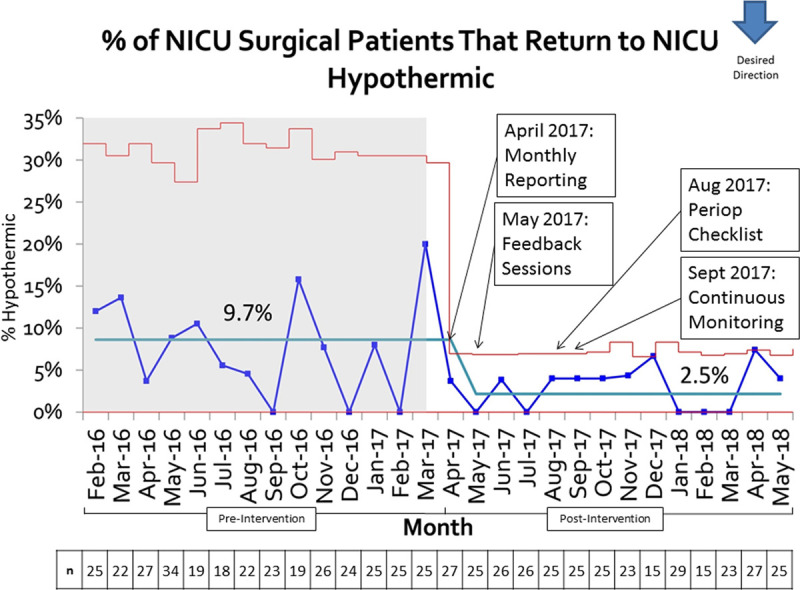
Control chart of % of NICU surgical patients that return to the NICU hypothermic. Red lines denote upper and lower confidence limits. The blue line denotes the actual incidence rate by month. The light blue line denotes the average incidence rate, including baseline rate and shift in average incidence rate. Green arrow at the right upper corner indicates goal direction. The goal of the quality improvement project is for the blue line to approach 0%. N = total number of NICU surgical patients per month.

While temperature data were collected in near real-time throughout the quality improvement project, the remaining data were then collected using a retrospective chart review of the electronic medical record from February 1, 2016 to May 30, 2018, for NICU patients undergoing procedures in the OR and returning to the NICU postoperatively. Inclusion criteria comprised all patients irrespective of age during this period, even patients who went to the OR during the intervention implementation phase. Exclusion criteria were patients undergoing cardiac procedures or procedures outside of the OR. Temperature data were tracked using a statistical control chart and evaluated using the IHI Model for Improvement Plan/Do/Study/Act (PDSA) cycles (Fig. 3). The Institutional Review Board approved this study as a quality improvement project.

### Measures

The primary outcome was the percentage of hypothermic patients (T < 36°C) on the first postoperative temperature. NICU nurses measured the temperature using a battery-operated digital temperature probe placed in the axilla immediately upon return from the OR to the NICU. No patients spent any time in the recovery room. The balancing measure was the number of hyperthermic patients (T > 37.9°C) on the first postoperative temperature. For 3 months following implementation, the team evaluated compliance with continuous axillary temperature monitoring of the infant during transport. The team used this compliance metric during the implementation phase of the axillary temperature monitoring intervention only. The team did not collect any other compliance data or process metrics.

Preoperative characteristics of patients collected included gestational age, postmenstrual age, sex, birth weight, weight at the time of surgery, American Society of Anesthesiologists (ASA) status, and the presence of a preoperative vasoactive medication. Intraoperative data included length of surgery, surgical specialty type, use of intraoperative vasoactive medications, need for transfusions, and intubation status upon return to the NICU.

### Analysis

The analysis compared the demographics and intraoperative characteristics between pre- and post-intervention groups using the Mann-Whitney U test for continuous data and Chi-square test and/or Fisher’s exact test (if any of the expected cell frequencies are <5) for categorical data. Summary statistics were presented as median with interquartile range and frequencies with percentages.

Multiple linear regression and multivariable Poisson regression with a robust variance estimate compare perioperative temperature measurements and incidence proportions of hypothermia between pre- and post-intervention groups.^17^ Confounders were included in the multivariable regression models if they were significant in the univariate analysis (Table 1). Q-Q plots of the residuals, the residuals versus the fitted values, and the residuals versus leverage tested the assumptions of multiple linear regression models. Shapiro-Wilks test and graphical assessments (histogram and Q-Q plot) checked the normality assumption.

**Table 1.**
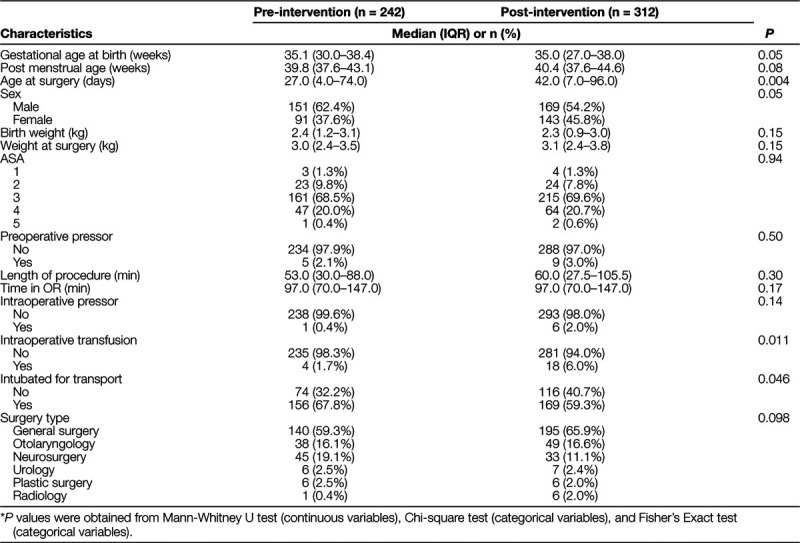
Demographics and Intraoperative Characteristics

All statistical tests were 2-sided and were performed at the 5% level of significance unless otherwise stated. Stata 15.1 software (StataCorp. 2017. Stata Statistical Software: Release 15. ; StataCorp LLC, College Station, TX) was used for all statistical analyses.

## RESULTS

From February 1, 2016 to May 30, 2018, data were collected for 554 patients (pre-intervention before April 1, 2017: 242 and post-intervention after April 1, 2017: 312). A comparison of the 2 groups did show a statistical difference in gestational age at birth (*P* < 0.05) and sex (*P* < 0.002) (Table 1). The pre-intervention group had a slightly higher gestational age at birth (35.1 weeks versus 35.0 weeks, *P* = 0.05) and a higher proportion of males (62.4% versus 54.2%, *P* = 0.004). The average age in days after birth (27.0 versus 42.0 days, *P* = 0.004) at the time of surgery was significantly lower in the pre-intervention group. Still, the postmenstrual age and weight of infants were not statistically different. A comparison of intraoperative characteristics between the pre-intervention group and post-intervention group showed a statistically significant difference in transfusion with fewer patients in the pre-intervention group receiving intraoperative blood products (1.7% versus 6.0%, *P* < 0.011) (Table 1). Another difference between the pre-intervention group and the post-intervention group was that post-intervention patients were more likely to be extubated at the end of the case (*P* < 0.047) (Table 1).

**Fig. 4.**
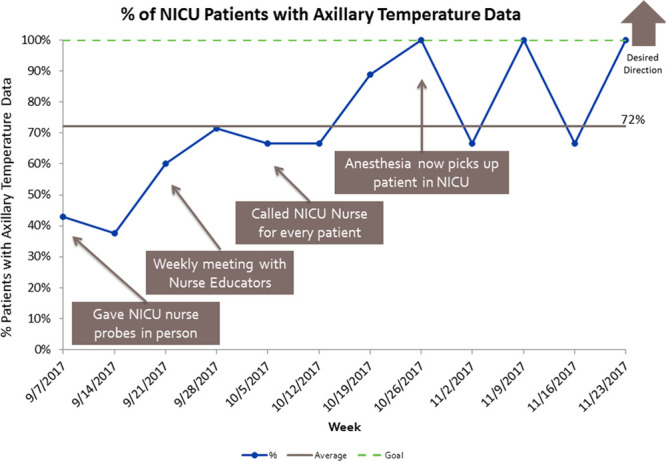
Chart of % of NICU patients with axillary temperature data.

The percentage of surgical patients who returned to the NICU hypothermic decreased from 9.7% (95% CI, 5.6%–13.8%) to 2.5% (95% CI, 0.7%–4.3%) (Table 2). The primary outcome decreased by 74% and produced a statistically significant shift due to achieving 8 data points below the baseline (Fig. 3). This change continued for greater than 12 months. The balancing measure, the incidence of postoperative hyperthermia, did not change during the study period. The average temperature of the patient immediately before leaving the NICU was 36.7°C (SD = 0.25°C). The mean time between when the patient left the OR and the first recorded temperature was 11.6 minutes (SD = 9.2 minutes). Anesthesia team compliance with continuous axillary temperature monitoring of the infant during transport was measured for 3 months from September 1, 2017 to November 30, 2017, following implementation and increased from 40% to 70% (Fig. 4).

**Table 2.**
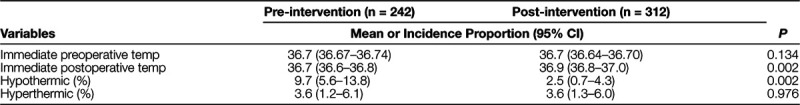
Perioperative Temperature Measurements

Postoperative hypothermia did not correlate with any demographic or intraoperative characteristics except for surgery type. The incidence of hypothermia in neurosurgical procedures, which often require significant skin exposure, was 3.6 times higher than general surgeries across the whole study period when pre-intervention and post-intervention periods were considered together (Incidence ratio: 3.6; 95% CI, 1.7–7.8; *P* = 0.001).

## DISCUSSION

The results show that a quality improvement project can reduce postoperative hypothermia in NICU surgical patients. This change continued over 12 months. Through active engagement and awareness of anesthesiologists, this project improved the temperature management of these patients. However, there were several confounders. While the average gestational age and postmenstrual age were not statistically different between the 2 groups, the patients in the post-intervention group were older based on age after birth (chronological age) by an average of 15 days. The difference in age at the time of surgery between the 2 groups mirrors trends in recent years to delay surgery. While heat production reaches adult levels (50 kcal/m^2^/h) by age 3–6 months when adjusted for weight and surface area, there are significant improvements in the infant’s ability to generate heat after 20 days of life, particularly in preterm infants.^18^ Hence, older infants in the post-intervention group may have had a better developed thermoregulatory system to maintain normothermia.

Another difference between the pre-intervention and post-intervention groups was the incidence of transfusions. Transfusion of warmed blood products may have increased body temperature, but this event occurred too infrequently to thoroughly explain the change in postoperative hypothermia prevalence in the post-intervention group. After reviewing cases where transfusion occurred in both groups, we could not identify a clear explanation for the increase in transfusion events found in the post-intervention group. There was no change in the clinical approach for intraoperative transfusion at our institution during this time. Surgical case mix and individual surgeons at our institution also remained stable. We did not evaluate estimated blood loss, and this metric is often not accurate.^19,20^ Finally, preoperative management of transfusion in the NICU did not change either.

There was a significant increase in NICU surgical patients who were extubated in the OR in the post-intervention group. Perhaps this trend is associated with differences in the gestational age and transfusion rates between the pre-intervention versus post-intervention groups. Infants with older gestational age and fewer intraoperative transfusions may be easier to extubate in the OR. Also, while not part of a deliberate quality improvement effort, this variation may also reflect changes in clinical practice to reduce the length of intubation and subsequently reduce the risk of ventilator-associated pneumonia, intensive care unit stay, and hospital stay as per recently published outcomes.^21–23^

We found that the anesthesia team compliance with continuous axillary temperature monitoring of the infant during transport increased from 40% to 70% over the 3 months from September to November following implementation. We attributed this to reminders about continuous monitoring during monthly reporting of incidence of hypothermia, the one-on-one meetings with anesthesia teams who had hypothermic patients, and NICU nursing interventions to ensure the temperature probe and monitoring cable were available and working.

Despite these confounders, there was a significant reduction in postoperative hypothermia with no change in hyperthermia incidence, indicating better temperature management of these surgical patients. While it is difficult to measure the impact of specific interventions, the pattern of the control chart (Fig. 3) suggests that monthly reporting of the percentage of hypothermic postoperative NICU patients had a significant impact because it was the first intervention implemented in April 2017, which coincided with a baseline shift. The baseline shift coincided with the implementation of individual feedback sessions for anesthesiologists who had cases of postoperative hyperthermia in May 2017. This intervention increased awareness and engagement among anesthesiologists. Further, feedback sessions allowed for the identification of additional interventions to improve temperature management. The implementation of the perioperative checklist in August 2017 and continuous temperature monitoring in September 2017 served to stabilize the reduction in postoperative hypothermia.

The higher incidence of postoperative hypothermia in neurosurgical procedures indicates that this is a particularly high-risk group. This observation is likely due to the significant skin exposure these procedures required during both the time of skin preparation and the surgery. During skin preparation, cool antiseptic is applied to a large area of the head and torso with the warming blanket off. During surgery, a significant portion of the infant’s surface area is in the surgical field and exposed to the cooler ambient OR environment.

### Limitations

There are several limitations to our project. While temperature measured at the axilla may be an accurate reflection of core body temperature in the NICU or emergency setting,^24^ it may be less accurate in the perioperative period because of the positioning of the patient, chemical warmer, or forced air blanket in the OR. Further, the timing of this postoperative measurement may vary depending on patient needs upon arrival to the NICU from the OR. Another limitation of this project is that we evaluated the temperature in this study as a single moment in time. The temperatures of the infant over the entire perioperative period would better reflect temperature control.

## CONCLUSIONS

The interventions used in this quality improvement project are effective in reducing postoperative hypothermia in NICU surgical patients. In particular, ongoing monitoring of postoperative hypothermia and reporting this outcome to all stakeholders will be key to sustaining this success. Supporting a culture wherein ongoing monitoring and feedback to anesthesiologists is routine and welcome is also essential to achieve normothermia in these infants.

Further quality improvement efforts can focus on improving temperature management in particularly vulnerable populations identified in this project, such as NICU patients undergoing neurosurgery. Additional work could also identify when neonates are vulnerable to developing perioperative hypothermia and the best interventions to target these periods and the impact of perioperative hypothermia on clinical outcomes for NICU surgical patients.

## DISCLOSURE

The authors have no financial interest to declare in relation to the content of this article.

## ACKNOWLEDGEMENTS

Assistance with the study: Reagan Grabowski, Rachel Emmett, Julius Brown, Joyce Doering, Dawn Brittingham, Lisa Zell.

## References

[1] Miller SS Lee HC Gould JB Hypothermia in very low birth weight infants: distribution, risk factors and outcomes. J Perinatol. 2011;31 Suppl 1 S49–S56. 21448204 10.1038/jp.2010.177

[2] Motoyama EK Davis P Smith’s Anesthesia for Infants and Children. 2006 7th ed, Philadelphia, Pa.: Mosby;

[3] Lyu Y Shah PS Ye XY; Canadian Neonatal Network. Association between admission temperature and mortality and major morbidity in preterm infants born at fewer than 33 weeks’ gestation. JAMA Pediatr. 2015;169:e150277. 25844990 10.1001/jamapediatrics.2015.0277

[4] Laptook AR Salhab W Bhaskar B; Neonatal Research Network. Admission temperature of low birth weight infants: predictors and associated morbidities. Pediatrics. 2007;119:e643–e649. 17296783 10.1542/peds.2006-0943

[5] Kurz A Sessler DI Lenhardt R Perioperative normothermia to reduce the incidence of surgical-wound infection and shorten hospitalization. Study of Wound Infection and Temperature Group. N Engl J Med. 1996;334:1209–1215. 8606715 10.1056/NEJM199605093341901

[6] Moslemi-Kebria M El-Nashar SA Aletti GD Intraoperative hypothermia during cytoreductive surgery for ovarian cancer and perioperative morbidity. Obstet Gynecol. 2012;119:590–596. 22353958 10.1097/AOG.0b013e3182475f8a

[7] Mahoney CB Odom J Maintaining intraoperative normothermia: a meta-analysis of outcomes with costs. AANA J. 1999;67:155–163. 10488289

[8] Schmied H Kurz A Sessler DI Mild hypothermia increases blood loss and transfusion requirements during total hip arthroplasty. Lancet. 1996;347:289–292. 8569362 10.1016/s0140-6736(96)90466-3

[9] Sessler DI Complications and treatment of mild hypothermia. Anesthesiology. 2001;95:531–543. 11506130 10.1097/00000542-200108000-00040

[10] Morehouse D Williams L Lloyd C Perioperative hypothermia in NICU infants: its occurrence and impact on infant outcomes. Adv Neonatal Care. 2014;14:154–164. 24824300 10.1097/ANC.0000000000000045

[11] American Society of Anesthesiologists. MIPS quality component. https://www.asahq.org/macra/qualitypaymentprogram/mips/mipsqualitycomponent19. Accessed September 3, 2019.

[12] Olmsted MG Powell R Murphy J Methodology: U.S. news & world report best children’s hospitals 2019-20. https://www.usnews.com/static/documents/health/best-hospitals/BCH_Methodology_2019-20.pdf. Accessed September 3, 2019.

[13] Frank SM Fleisher LA Breslow MJ Perioperative maintenance of normothermia reduces the incidence of morbid cardiac events. A randomized clinical trial. JAMA. 1997;277:1127–1134. 9087467

[14] Vinci A Islam S Quintos-Alegheband L A quality improvement intervention to decrease hypothermia in the delivery room using a checklist. Pediatr Qual Saf. 2018;3:e125. 31334457 10.1097/pq9.0000000000000125 PMC6581478

[15] Engorn BM Kahntroff SL Frank KM Perioperative hypothermia in neonatal intensive care unit patients: effectiveness of a thermoregulation intervention and associated risk factors. Paediatr Anaesth. 2017;27:196–204. 27917566 10.1111/pan.13047

[16] Brozanski BS Piazza AJ Chuo J STEPP IN: working together to keep infants warm in the perioperative period. Pediatrics. 2020;145:e20191121. 32193210 10.1542/peds.2019-1121

[17] Zou G A modified Poisson regression approach to prospective studies with binary data. Am J Epidemiol. 2004;159:702–706. 15033648 10.1093/aje/kwh090

[18] Roberton NRC Textbook of Neonatology. 1986 Edinburgh; New York, N.Y.: Churchill Livingstone;

[19] Rothermel LD Lipman JM Estimation of blood loss is inaccurate and unreliable. Surgery. 2016;160:946–953. 27544540 10.1016/j.surg.2016.06.006

[20] Yoong W Karavolos S Damodaram M Observer accuracy and reproducibility of visual estimation of blood loss in obstetrics: how accurate and consistent are health-care professionals? Arch Gynecol Obstet. 2010;281:207–213. 19434419 10.1007/s00404-009-1099-8

[21] Alam S Shalini A Hegde RG Predictors and outcome of early extubation in infants postcardiac surgery: a single-center observational study. Ann Card Anaesth. 2018;21:402–406. 30333334 10.4103/aca.ACA_209_17 PMC6206803

[22] Abuchaim DC Bervanger S Medeiros SA Early extubation in the operating room in children after cardiac heart surgery. Rev Bras Cir Cardiovasc. 2010;25:103–108. 20563475 10.1590/s0102-76382010000100020

[23] Chae MS Kim JW Jung JY Analysis of pre- and intraoperative clinical for successful operating room extubation after living donor liver transplantation: a retrospective observational cohort study. BMC Anesthesiol. 2019;19:112. 31248376 10.1186/s12871-019-0781-z PMC6598245

[24] Friedrichs J Staffileno BA Fogg L Axillary temperatures in full-term newborn infants: using evidence to guide safe and effective practice. Adv Neonatal Care. 2013;13:361–368. 24042144 10.1097/ANC.0b013e3182a14f5a

